# Wilson’s disease in pregnancy: case series and review of literature

**DOI:** 10.1186/1756-0500-6-421

**Published:** 2013-10-18

**Authors:** Ayesha Malik, Ali Khawaja, Lumaan Sheikh

**Affiliations:** 1 Department of Obstetrics and Gynecology, The Aga Khan University Hospital, Stadium Road, P.O. Box 3500, Karachi 74800, Pakistan; 2 Medical Student, Medical College, The Aga Khan University Hospital, Stadium Road, P.O. Box 3500, Karachi 74800, Pakistan

**Keywords:** Copper metabolism, Infertility, Pregnancy, Wilson’s disease, Zinc sulphate

## Abstract

**Background:**

Wilson’s disease is a rare, autosomal recessive inherited disorder characterized by impaired liver metabolism of copper leading to decreased biliary excretion and incorporation of ceruloplasmin levels mainly in the liver and brain. Untreated Wilson’s disease has been shown to cause subfertility and even in cases where pregnancy occurs, it often results in spontaneous miscarriage.

**Case presentations:**

We present four cases of successful pregnancy outcomes in three patients diagnosed with Wilson’s disease along with the literature review. All the patients were managed with zinc sulphate without any postnatal complications.

**Conclusion:**

Patients with Wilson’s disease receiving regular treatment who remain asymptomatic are usually able to conceive and achieve successful outcomes. However, these pregnancies should be considered high risk and merit regular surveillance.

## Background

Wilson’s disease is a rare autosomal recessive disorder with a prevalence of 1:50,000-1:100,000 live births [1]. Mutation of ATP 7B on chromosome 13q14 leads to impaired biliary excretion and ceruloplasmin incorporation causing copper accumulation mainly in the liver and brain [2]. This accumulation results in liver cirrhosis and nervous system manifestations such as movement disorders and ataxia [3].

Untreated Wilson’s disease usually causes subfertility and in cases where pregnancy does occur, it often results in spontaneous miscarriage [4]. However, therapeutic evolution in the past decades has resulted in multiple successful pregnancy outcomes in patients with Wilson’s disease [3-8]. Penicillamine, zinc salts, trientine and tetrathiomolybdate have been shown to be highly efficacious in treating this disorder [1].

We report four cases of successful pregnancy outcomes in three women with Wilson’s disease (all treated with zinc) together with the literature review regarding the impact of Wilson’s disease on fetomaternal outcomes.

## Case presentations

### Case 1

A 30 year old lady presented to our antenatal clinic during the first trimester of her pregnancy. She was diagnosed with Wilson’s disease at the age of twenty two while being investigated for involuntary generalized shaking of the body. Her laboratory investigations revealed increased level of copper in the urine and low serum ceruloplasmin. She was prescribed Zinc Sulphate 50 mg twice daily subsequently. Her brother and a sister were also diagnosed to have Wilson’s disease. She had previously conceived on clomiphene after two years of subfertility but underwent two first trimester miscarriages and a stillbirth at 30 weeks of pregnancy due to preeclampsia and placental abruption.

During her initial presentation, the patient complained of headache and tremors for which the neurology team was consulted. The dose of zinc sulphate was increased to 50 mg thrice a day after which the neurological symptoms subsided gradually. Ophthalmologic examination revealed Kayser-Fleischer rings. Serum copper and ceruloplasmin levels were then obtained which were 76.5 μg/dL (118–302 μg/dL) and 0.03 g/L (0.25-0.63 g/L), respectively. Maternal echocardiogram was performed which was within normal limits while ultrasound of the upper abdomen revealed inflammatory changes in the liver with echogenicity of the parenchyma. An anomaly scan was performed at 20 weeks of gestation which did not reveal any congenital anomaly while a growth scan performed at 35 weeks of gestation showed a live fetus weighing 2.5 kg and having adequate amniotic fluid index. Renal and liver function tests remained within normal limits throughout the pregnancy. Serum copper level was again obtained at 30 weeks of gestation which was 30.72 μg/dL.

The patient underwent spontaneous labour at 37 weeks of gestation. Intra-partum course of events was unremarkable and a healthy baby boy weighing 3 kg with a good Apgar score was delivered vaginally.

### Case 2

A 33 year old lady with a prior history of a missed miscarriage, presented to our antenatal clinic at six weeks of gestation. She was diagnosed to have Wilson’s disease four years ago while undergoing evaluation for muscle weakness. The urinary copper level was high while serum copper and cerulopasmin levels were found to be below normal limits and she was started on zinc sulphate three times a day.

The patient had conceived spontaneously and was asymptomatic at the time of presentation. Zinc sulphate was continued at 30 mg thrice a day throughout her pregnancy. As a part of her baseline workup, an ultrasound upper abdomen was performed which showed portal hypertension. Serum copper level was 33 μg/dL while serum ceruloplasmin was 0.05 g/L. The blood pressure during her first antenatal visit was within normal limits. However, it was found to be raised (150/100 mmHg) at 18 weeks of gestation due to which methyldopa 500 mg three times a day was started subsequently. Blood pressure on her following visits remained between 130/80 mmHg and 140/90 mmHg. Anomaly scan performed at 22 weeks of gestation did not reveal any congenital anomaly. At 24 weeks of gestation, 50 grams glucose challenge test was performed for screening of gestational diabetes mellitus which was found to be deranged; one hour value of 177 mg/dl (normal value = 140 mg/dl). Oral glucose tolerance test was therefore performed which also revealed raised blood glucose levels confirming the diagnosis of gestational diabetes mellitus. Her sugars, however, remained well controlled through dietary measures throughout the antenatal period. A growth scan was performed at 34 weeks of gestation which showed a live fetus with an estimated fetal weight of 2.1 kg with normal amniotic fluid index. Serum copper and ceruloplasmin levels performed during this time period were 70.86 μg/dL and 0.07 g/L, respectively. During her 38th week of pregnancy, she developed preeclampsia with a blood pressure of 140/95 mmHg and proteinuria of 0.3 mg/dl. Therefore, induction of labour was offered. The bishop score prior to the induction was four and cervical ripening was performed through an intra-cervical foley’s catheter followed by an intra-vaginal prostaglandin E2. Syntocinon was later started at a cervical dilatation of 3 cm. However, an emergency caesarean section had to be performed due to non-progress of labour. An alive baby boy weighing 3.3 kg with a good Apgar score was delivered successfully. Her blood pressure normalized during the postpartum period. One year later, the patient had another spontaneous pregnancy which terminated in a missed miscarriage at 7 weeks of gestation.

### Case 3

A 21 year old primigravida, presented to our antenatal clinic for her booking appointment. She was diagnosed to have Wilson’s disease at the age of eighteen years when she started having symptoms of depression. The laboratory workup revealed an elevated level of copper in the urine (250 μg/day; normal value <60 μg/day) and low serum ceruloplasmin level (0.1 g/l; normal range 0.25-0.63 g/l). Her father was also diagnosed to have Wilson’s disease. She had been using zinc sulphate 60 mg three times a day and relaxipam 2 mg daily since the time of diagnosis and continued it throughout her pregnancy.

The patient did not have any symptoms of Wilson’s disease at the time of presentation. All the antenatal laboratory tests were within normal limits. Urinary copper level was obtained at the tenth week of gestation which was 2.88 μg/day. Anomaly scan performed at 20 weeks revealed no gross congenital anomalies while the growth scan done at the 30th week of gestation showed satisfactory growth of the fetus. Maternal echocardiography performed at 24 weeks did not reveal any abnormality with an ejection fraction of 60%. Oral glucose tolerance test was obtained at the 24th week of gestation which ruled out gestational diabetes.

Antenatal course was uneventful except for the two episodes of gastroenteritis at the 23rd and 36th week of gestation for which she was admitted and managed conservatively.

At 40 weeks of gestation, she was admitted electively for the induction of labor. Bishop score at the time of admission was six. The induction of labour was initiated with an intracervical foley’s catheter followed by an intravaginal prostaglandin E2. Amniotomy was subsequently performed followed by intravenous oxytocin once the contractions started. During her intrapartum monitoring, blood pressure was found to be escalating and due to a persistently raised diastolic pressure of 100 mmHg, an intravenous hydralazine was administered. Spot protein/creatinine ratio obtained during this time period was 0.33 mg/dl which was suggestive of preeclampsia. However, labour progressed satisfactorily and the patient delivered an alive baby boy weighing 3.4 kg with a good Apgar score via spontaneous vaginal delivery. In the post partum period, blood pressure remained within normal limits on 5 mg of amlodipine.

### Case 4

The same patient conceived spontaneously one year later. Her antenatal visits were regular and unremarkable. She was asymptomatic for Wilson’s disease throughout the pregnancy. Serum ceruloplasmin levels were checked at 24 and 36 weeks of gestation which were 0.73 and 0.75 g/L, respectively. Anomaly and Growth scans were unremarkable.

At 38 weeks of gestation, she presented with complains of abdominal heaviness, stiffness, vomiting and raised blood pressure of 140/100 mmHg. Urinalysis was negative for proteinuria while full blood count, liver function tests and uric acid were within normal limits. She was admitted and planned for induction of labor.

Intrapartum period remained uneventful culminating in a spontaneous vaginal delivery of a healthy baby boy weighing 3.5 kg. Patient remained normotensive in the postpartum period and was discharged on the second postnatal day.

In all our patients, serum ceruloplasmin level was ascertained by nephlometric method while urinary and serum copper levels were determined by FASS (flame atomic absorption spectrometry) methodology.

## Discussion

Although there are a few case reports pertaining to successful pregnancy outcomes in women with Wilson’s disease [3-8], the present case series enforces the effectiveness of zinc sulphate in managing pregnancies in patients diagnosed with Wilson’s disease.

Liver is the primary storage organ for copper after which it can be distributed in circulation to other tissues such as nervous system, eyes and kidneys. Accumulation of excessive copper due to decreased excretion can adversely affect these tissues leading to hepatic injury and cirrhosis, neurological symptoms such as dyskinesias and tremors, kayser-fleishcer ring around limbus and renal tubular damage [9]. Moreover, it can lead to menstrual irregularities due to hepatotoxicity and recurrent miscarriages due to copper deposition in the uterus in women in reproductive age group [1,4]. Women with Wilson’s disease may require infertility treatment but many patients conceive spontaneously. In our case series, one patient received clomephine citrate. However, women with severe liver disease leading to bleeding episodes from eosophageal varices or hepatic insufficiency are usually discouraged from getting pregnant [8].

The serum copper and ceruloplasmin levels have been observed to change as the pregnancy progresses. The levels may increase till 24 weeks followed by a modest decline probably due to fetal intake of copper [7]. There is approximately 12 mg of copper in a neonate and the fetus is thought to remove an average of 0.044 mg of copper per day from the maternal serum, due to which improvement in symptoms of Wilson’s disease have also been reported [7,10].

Untreated, Wilson’s disease may lead to early pregnancy complications [4]. Two out of three of our patients had at least one miscarriage. The mechanism has been postulated to be similar to that of copper containing contraceptive devices which exert their contraceptive effect by causing deposition of copper in the endometrium giving rise to an excess of copper ions [11]. Such high concentrations of intrauterine copper may lead to miscarriages as seen in patients with Wilson’s disease.

Wilson’s disease, if not treated promptly, can lead to significant morbidity and can be potentially fatal. It is hence imperative that it is identified early and treated effectively. Safety of Pencillamine in managing Wilson’s disease during pregnancy has been reported by multiple studies [4-6,8]. Only one case report states an infant developing connective tissue disorder in a mother with a daily intake of 2 grams of pencillamine which is approximately twice the dose used for Wilson’s disease in pregnancy [12]. It has also been suggested to reduce the dose of pencillamine to 0.25 grams per day, one to six weeks prior to a caesarean section in order to prevent delayed wound healing [13-15]. Trientine is an oral chelating drug used as an alternate when patients develop a reaction to pencillamine. Its effectiveness has been proven and case reports regarding its use in pregnancy do not show any adverse fetomaternal outcomes [16,17].

More recently, zinc is increasingly being used as a therapeutic option in managing Wilson’s disease. Zinc interferes with the absorption of copper from the gastrointestinal tract; however, its long-term side-effects are still not well studied. Zinc performs its function by induction of intestinal cells metallothionein which has a high affinity for copper and prevents serosal transfer of copper into blood [18]. Brewer et al. reports use of Zinc Sulphate in 26 pregnancies out of which 24 had a normal infant, one infant had a congenital heart defect and one had microcephaly [10]. In our case series, all the patients were treated with zinc sulphate without occurrence of any congenital anomaly in the neonates.

In our case series, three out of four pregnancies were complicated with pregnancy induced hypertension/preeclampsia. This complication has been reported previously in patients with Wilson’s disease [1,19]. It may be hypothesized that these patients are at risk of developing pregnancy induced hypertension or preeclampsia. However, it is still unknown if this finding is related to the presence of the underlying disease or is related to the anti-copper therapy used during pregnancy. These patients might be at risk for other complications including placental abruption and thrombocytopenia and deranged coagulation [3,19,20]. However, such complications were not encountered in our patients.

## Conclusions

Patients with Wilson’s disease receiving regular treatment who remain asymptomatic are usually able to conceive with successful outcomes. Zinc Sulphate is an effective therapeutic option and can be safely used in managing patients with Wilson’s disease throughout the pregnancy. However, since there seems to be an association of Wilson’s disease with miscarriages and pregnancy induced hypertension/preeclampsia, these pregnancies should be considered high risk and merit regular surveillance.

## Consent

Written informed consent was obtained from the patients for publication of this Case Series. A copy of the written consent is available for review by the Editor-in-Chief of this journal.

## Competing interests

The authors declare that they have no competing interests.

## Authors’ contributions

AM followed the patients regularly, collected the required data and contributed in manuscript writing. AK contributed towards data collection and manuscript writing. LS identified the patients and critically edited the manuscript. All authors read and approved the final manuscript.
